# Sesquiterpene Lactones from *Artemisia absinthium*. Biotransformation and Rearrangement of the Insect Antifeedant 3α-hydroxypelenolide

**DOI:** 10.3390/plants10050891

**Published:** 2021-04-28

**Authors:** Braulio M. Fraga, Carmen E. Díaz, María Bailén, Azucena González-Coloma

**Affiliations:** 1 Instituto de Productos Naturales y Agrobiología, CSIC, Avda. Astrofísico F. Sánchez 3, 38206 La Laguna, Tenerife, Canary Islands, Spain; bmfraga@telefonica.net; 2 Departamento de Medicina Preventiva y Salud Pública y Microbiología, Facultad de Medicina, Universidad Autónoma de Madrid, C. Arzobispo Morcillo s/n, 28049 Madrid, Spain; maria.bailen@uam.es; 3 Instituto de Ciencias Agrarias, CSIC, Serrano 115 dpdo., 28006 Madrid, Spain

**Keywords:** *Artemisia absinthium*, asteraceae, absilactone, hansonlactone, ajenjol, *Mucor plumbeus*, biotransformation, 3α-hydroxypelenolide, biological activity

## Abstract

Three new compounds, the sesquiterpenes absilactone and hansonlactone and the acetophenone derivative ajenjol, have been isolated from a cultivated variety of *Artemisia absinthium*. In addition, the major lactone isolated, 3α-hydroxypelenolide, was biotransformed by the fungus *Mucor plumbeus* affording the corresponding 1β, 10α-epoxide. A cadinane derivative was formed by an acid rearrangement produced in the culture medium, but not by the enzymatic system of the fungus. Furthermore, 3α-hydroxypelenolide showed strong antifeedant effects against *Leptinotarsa decemlineata* and cytotoxic activity to Sf9 insect cells*,* while the biotransformed compounds showed antifeedant postingestive effects against *Spodoptera littoralis.*

## 1. Introduction

The *Artemisia* genus with more of 400 species is one of the largest genera in the Asteraceae family. The species *Artemisia absinthium* L., known as wormwood, is a perennial medicinal species which has been widely investigated due to its ethno-pharmacological interest.

*A. absinthium* is abundant in the mountains of Spain as a ruderal species. There are seven chemotypes described in the Iberian Peninsula [1]. A thujone-free population of this plant (*A. absinthium* var. *candial*) has been domesticated for the production of essential oil [2]. The oil characterized by the presence of *cis*-epoxyocimene, (−)-*cis*-chrysanthenol, chrysanthenyl acetate, linalool and trans-caryophyllene showed strong antifungal effects [2]. Furthermore, two ocimene monoterpenes were isolated and their absolute configurations determined by vibrational circular dichroism (VCD) [3].

However, the non-volatile constituents of *A. absinthium* have received little attention. This plant is characterized by its content in the sesquiterpene lactones hydroxypelenolide (**3**) and ketopelenolides A and B (**4**–**5**) [4,5,6]. Additionally, five sesquiterpene lactones, a flavone [7] and a germacrane lactone artabolide with auxin transport inhibitor properties [8] have been also reported. A previous study on the domesticated *A. absinthium* var. *candial* reported the lactone hydroxypelenolide (**3**), with insect antifeedant effects, and the flavones artemetin and casticin [6] as the main components of its non-volatile extract [6].

Biotransformations of terpenoids can modify the biological activities of the starting compounds [9] and are also used to study structure-activity relationships of bioactive compounds [10]. In this context, *Mucor plumbeus*, with a broad substrate specificity [11,12], has been used in biotransformations of diterpenes to develop models to explain their hydroxylations [13,14,15,16,17] and for the biotransformation of an africanane sesquiterpene to give epoxy derivatives with improved insect antifeedants and insecticidal effects [18]. In this work we describe three unknown compounds, the sesquiterpene lactones absilactone (**1**) and hansonlactone (**2**) and the acetophenone derivative artenol (**12**) [4], isolated from *A. absinthium* var. *candial* along with several known compounds. Furthermore, 3α-hydroxypelenolide (**3**), previously known as hydroxypelenolide [4], was biotransformed by *Mucor plumbeus* to give 1β, 10α-epoxide (**9**) and a cadinane derivative (**10**). The latter was formed by an acid rearrangement produced in the culture medium, not by the enzymatic system of the fungus. We also describe the insecticidal activities of these compounds against the insect pests *Spodoptera littoralis*, *Myzus persicae*, the cytotoxic effects on *Spodoptera frugiperda* pupal ovarian tissue cells (Sf9), and the phytotoxic effects on *Lactuca sativa*.

## 2. Results and Discussion

### 2.1. Components of A. absinthium

Absilactone is a new *nor*-sesquiterpene, which structure has been determined as **1** based on the following considerations: Its high-resolution mass spectrum showed the molecular ion at *m/z* 248.1040, which corresponds to the molecular formula C_14_H_16_O_4_. In the ^1^H-NMR spectrum three methyl groups were observed, two of which are over double bonds, resonating as singlets at *δ*_H_ 2.23 and 2.36, while the third corresponds to a secondary methyl that appears as a doublet at *δ*_H_ 1.25 (*J* = 7.0 Hz). The geminal proton (H-6) at the lactonic ring closure resonates at *δ*_H_ 4.85. Its coupling constant with H-7 (10.6 Hz), indicated a trans-relationship between these H-6 and H-7 hydrogens (Figure S1).

The ^13^C NMR spectrum showed, in addition to the three methyl groups, two methylenes at *δ*_C_ 26.0 and 37.6, the relatively low value of the first is typical of being located between another methylene and a methine, so it was assigned to C-8. The methine group at C-11 was observed at *δ*_C_ 41.5. Other signals present in this spectrum were six singlets, two of them at *δ*_C_ 166.8 and 177.6 characteristic of the carbonyl groups of the two lactones, while the other four singlets correspond to carbons of the two tetrasubstituted double bonds (Figure S2).

The proposed relative structure for this compound **1** was confirmed considering two-dimensional NMR spectra. Thus, in the HMBC experiment, the following correlations were observed: H-6 with C-4/C-5/C-8; H-7 with C-6; H-8 with C-6/C-7/C-9; H-9 with C-1/C-7/C-8,C-10; H-11 with C-7/C-8/C-12/C-13; H-13 with C-7/C-11/C-12; H-14 with C-1/C-9/C-10 and H-15 with C-4/C-5. The NOESY experiment allowed determining the β-stereochemistry for H-11, since this hydrogen correlates with H-6β and H-8β. Other observed connectivities were the H-13 methyl with H-7α/H-8α/H-11, and H-8α with H-9β.

Austroyunnane F (**7**) is a *nor*-sesquiterpene of the absilactone type, which was isolated from *A. austro-yunnanensis* [19]. This compound and absilactone (**1**) probably derive from a similar biosynthetic pathway, considering that both possess a similar framework and have been obtained from species of the *Artemisia* genus. Another analogous *nor*-sesquiterpene but with a pseudoguaiane skeleton, 4-hydroxy-*nor*-psilotropin (**8**), has been obtained from *Psilotrophe villosa* [20].

A new sesquiterpenoid with an eudesmanolide structure has also been isolated from *A. absinthium* and named hansonlactone (**2**). Its HRMS was in accordance with the molecular formula C_15_H_18_O_4_ (*m/z* 262.1212). The ^1^H NMR spectrum showed three methyl groups, one angular at *δ*_H_ 1.26 (H-14), another secondary at 1.20 (d, *J* = 7.0 Hz, H-13) and a third situated over a tetrasubstituted double bond at 2.09 (d, *J* = 1.5 Hz, H-15). This last methyl is coupled with H-3, a proton of another double bond at *δ*_H_ 5.69 (dd, *J* = 7, 1.5 Hz), which in turn is coupled with H-2 at 6.37 (d, *J* = 7 Hz). The resonance at *δ*_H_ 5 (1H, dt, *J* = 11.8, 1.9 Hz) was originated by the geminal proton (H-6) to the lactone ring closure, which showed a trans-axial coupling with H-7 (*J* = 11.8 Hz). The ^13^C NMR spectrum showed the corresponding signals of the 2,3- and 4,5- double bond at *δ*_C_ 138.8/117.3 and 127.9/129.6, respectively (Figures S3 and S4). The carbonyl groups of the two lactones resonate at *δ*_C_ 168.8 (C-1) and 178.4 (C-12). In the HMBC experiment correlations were observed of H-2 with C-1/C-3/C-4; H-3 with C-2/C-5/C-15; H-8 with C-6/C-7/C-9/C-10/C-11; H-9 with C-1/C-7/C-8/C-10/C-14 and H-11 with C-13, which confirmed the structure **2**. The assigned configuration at C-6, C-7 and C-11 was based in the NOESY experiment, with correlations of H-6 with H-14 and of H-7 with H-11, and also considering the coupling of H-6 with H-7 (*J* = 11.8 Hz) indicated above. Other NOESY crosspeaks were due to H-2 with H-3; H-3 with H-15; H-7 with H-8α H-9β with H-14; and H-11 with H-13.

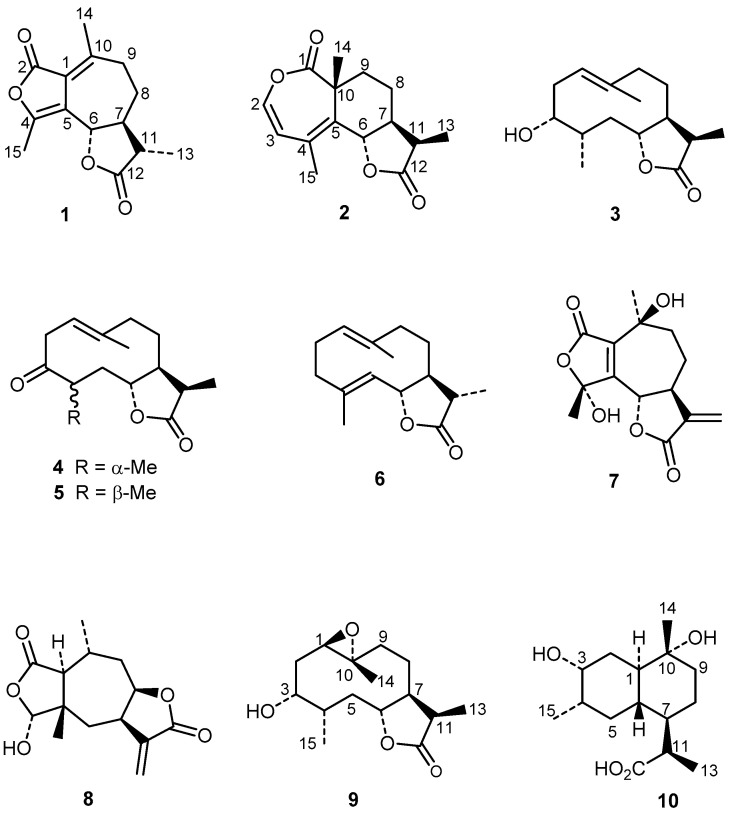



Another undescribed compound isolated from this plant was an acetophenone derivative, which we have named ajenjol. Its structure **12** was assigned considering its spectroscopic data: In the HRMS the molecular ion appears at *m/z* 250.1201, which corresponds to the molecular formula C_14_H_18_O_4_. The ^1^H NMR spectrum showed resonance of the two aromatic protons as singlets at *δ*_H_ 6.45 and 8.25, the former located between carbons bearing oxygens and the latter situated in ortho position to the acetyl group. Signals of four methyl groups were observed, two from the side chain at *δ*_H_ 0.95, one from the acetyl group at *δ*_H_ 2.62 and another from the methoxy group at *δ*_H_ 3.94. A doublet of the two H-10, located on a carbon in α-position to a carbonyl group, appears at *δ*_H_ 2.81, and the H-11 methine resonates as a double triplet at *δ*_H_ 2.21. A singlet at *δ*_H_ 12.92 was due to the hydroxylic hydrogen bonded with the carbonyl of the acetyl group (Figure S13).

The ^13^C-NMR spectrum confirmed the presence of the aromatic ring, with resonances of two doublets at *δ*_C_ 99.8 and 135.2, located between carbons bearing oxygens and carbonyl groups, respectively, and four singlets to *δ*_C_ 113.8, 121.0, 164.8 and 167.5. The last two carbons linked to methoxy and hydroxy groups, respectively. The two carbonyl groups appeared in this spectrum at *δ*_C_ 199.5 and 203.4, the latter corresponding to the acetyl group (Figure S14). In the HMBC spectrum, the following connectivities were observed: H-3 with C-1/C-2/C-5; H-6 with C-2/C-4; H-8 with C-7; H-11 with C-9/C-12/C-13; H-12/H-13 with C-10; -OH with C-1 and H-14 (-OMe) with C-4. In the NOESY experiment, the main correlations detected were H-3 with the methoxy group (H-14) and H-6 with the methyl (H-8) and the methylene (H-10). Structures **12** and **13** met the data indicated above. Ajenjol was assigned to structure **12** on the basis of the following considerations: We have now described and assigned the ^13^C NMR spectrum (Figure S12) of espeletone (**11**) [21], which values of C-10. C-11, C-12 and C-13 in its side chain and those of ajenjol (**12**) were identical (Table 1). This fact indicated that both compounds have a methoxy group at the C-4 position, which was confirmed observing that the resonance values above indicated for **11** and **12** were different for the corresponding carbons in **14**. This last product, isolated from *Polymnia sonchifolia* [22], has a hydroxyl group at C-4 with the hydrogen bonded to the C-9 carbonyl, affecting the carbon resonances of the side chain, especially to C-10. The reported ^13^C NMR data of **14** have been assigned here (Table 1). Structure **12** of ajenjol, and not **13**, was also in accordance with the chemical shift of the C-7 carbonyl. Thus, this carbon appears in **12** at *δ*_C_ 203.4, a value more similar to that of 6-hydroxytremetone (**15**) (*δ*_C_ 201.9) [23] than that of 6-methoxytremetone (**16**) (*δ*_C_ 197.9) [24]. On the other hand, espeletone (**11**), which we have now also isolated from *A. absinthium*, could be a possible biogenetic precursor of this new compound, ajenjol (**12**).

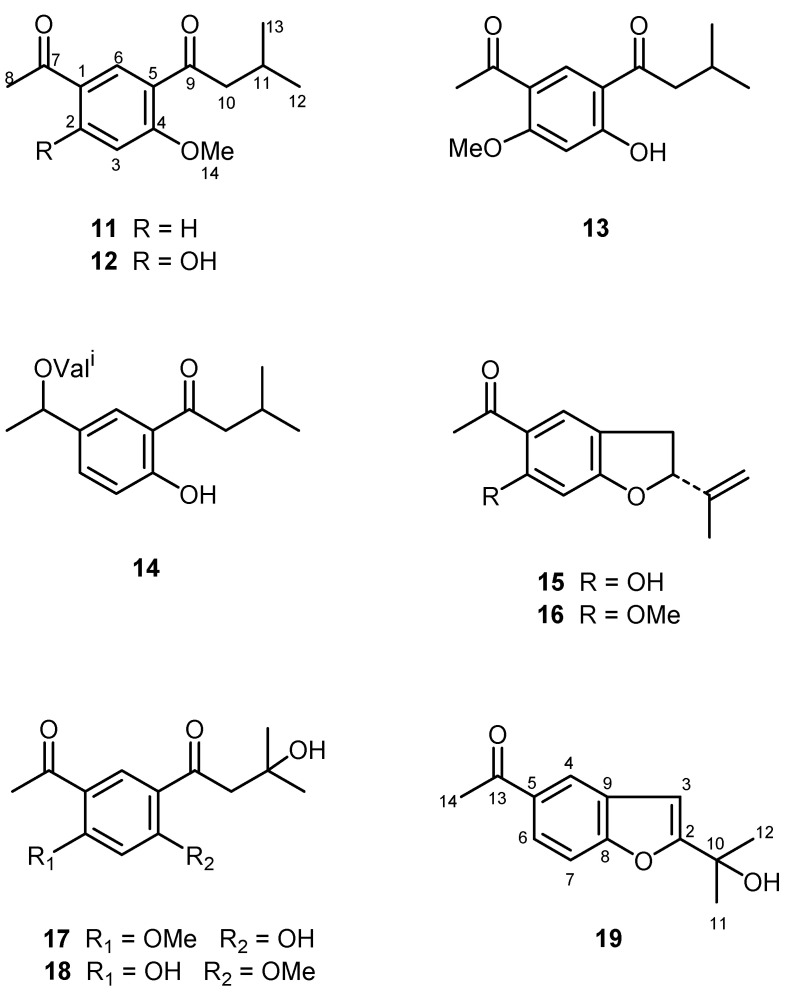



Structure **17**, with an OH group at C-4, had been assigned to glutinosol, which was obtained from *A. glutinosa* [25]. Unfortunately, the ^13^C NMR data for this compound were not reported, and the ^1^H NMR spectrum described can be valid for **17** but also for **18**. Indeed, the presence of espeletone (**11**) in this plant, and its 10, 11-dehydro derivative, indicates that 1**8** could be an alternative structure.

Additional known compounds isolated from *A absintium* have been the monoterpene (-)-(3*S*, 5*Z*)-2,6-dimethylocta-5,7-dien-2,3-diol [2,3,26], the sesquiterpene lactones 3α-hydroxypelenolide (**3**) [4,5,6], ketopelenolide A (**4**) [4], ketopelenolide B (**5**) [4,27], and dihydrocostunolide (**6**) [27], the diterpene dehydroabietic acid [28], the triterpenes 24-methylencycloartanol and its acetate [29], lagerenol and its acetate [30], and cycloart-23(*Z*)-en-3β,25-diol [31], the acetophenone espeletone (**11**) [21], a benzofurane derivative (**19**) [22,32] and the flavones artemetine [33,34] and casticine [35]. We have now assigned the ^1^H and ^13^C NMR spectra of espeletone (**11**) (Figures S11 and S12) and of the benzofuran derivative **19** (Figures S15 and S16) using 2D NMR data (Table 1).

### 2.2. Biotransformation and Rearrangement of 3α-hydroxypelenolide (***3***)

During the past years we have been interested in the microbiological transformation of diterpenes by the fungus *M. plumbeus* [13,14,15,16,17]. The aim of these studies has been to develop models to explain the hydroxylation of these compounds by this microorganism, which possesses a broad specificity in the substrate [11,12]. To complement these works, in order also to study their structure-activity relationship as potential pesticides, we expand this research to the sesquiterpenes. Thus, we have investigated the biotransformation of an africanane sesquiterpene by this fungus [18]. Now, continuing with these studies, and considering the good yield of 3α-hydroxypelenolide isolated from *A. absinthium*, we have biotransformed it by the fungus *M. plumbeus* affording two products **9** and **10**.

The less polar of the compound isolated was **9**, which showed a ^1^H NMR spectrum with very broad signals and a ^13^C NMR with few resonances. These effects are due to the conformational flexibility that possesses the 10-membered ring of some germacranolides at ambient temperature (298 °K). To obtain these spectra in better conditions we ran them at 233 °K, although in fact at this temperature very small couplings could not be observed. Their NMR spectra, in comparison with that of substrate (Figures S5 and S6), showed lack of the double bond resonances, which were substituted by the corresponding to an oxirane ring at *δ*_H_ 3.28 (H-1) and *δ*_C_ 58.7 (C-1) and 60.3 (C-10) (Figures S7 and S8). Thus, in the HMBC experiment, connectivities were observed of H-1 with C-2/C-3; H-2 with C-1/C-10; H-9 with C-10; and H-14 with C-1/C-10. The relative low field resonance at *δ*_H_ 3.28 of H-1, the geminal proton to the epoxy group, was due to the presence of the 3α-OH, which also permitted H-1 to be assigned an α-stereochemistry. The geminal methyl (C-14) to the epoxide at C-10 was given a β-stereochemistry, because it showed a crosspeak with H-6 in the NOESY experiment. Thus, the epoxide ring has a trans-geometry as also had the double bond of the substrate. The chair-chair conformation of the 10-membered ring at 233 °K was determined also considering NOESY data. Thus, correlations of H-6 with H-4/H-8β/H-14 indicated that they have an axial configuration in the β-face of the molecule, whilst correlations of H-1 with H-7/H-9α showed that these hydrogens were located in the α-face. This molecular conformation was confirmed by computational analysis, and resulted similar to that described for another 1β, 10α-epoxy-11, 13-dihydrogermacranolide, isolated from *Achillea crithmifolia* [36,37].

**Figure 1:**
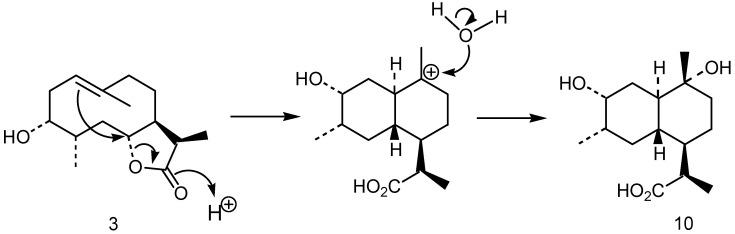
Rearrangement of 3α-hydroxypelenolide (**3**) into a cadinane derivative (**10**).

Compound **10**, also obtained in the fermentation of the substrate **3**, was not formed by the enzymatic system of *M. plumbeus*, but by acid rearrangement of **3** in the culture medium (Figure 1). This fact was confirmed in another experiment carried out under the same conditions but in the absence of the fungus, where the incubation of compound **3** did not afford the epoxide **9**. The structure **10** assigned to this product was based in the following considerations: Its HRMS showed the fragment of higher mass at *m/z* 255.1596 (C_14_H_23_O_4_), which is formed from the molecular ion by loss of a methyl group. Thus, its molecular formula was C_15_H_26_O_4_. In the NMR spectra does not appear the resonances of the double bond and the geminal proton to the lactone closure, which have taken part in the rearrangement, being substituted by a methylene group at *δ*_H_ 1.22 and 2.10 (*δ*_C_ 34.2, C-2), a methine at *δ*_H_ 1.29 (δ_C_ 42.1, C-6), and a tetrasubstituted carbon bearing a hydroxy group at *δ*_C_ 72.6 (C-10). The carbon resonance of the acid group, formed by opening of the lactone, appears at *δ*_C_ 179.3 (Figures S9 and S10). The corresponding HMBC connectivities were H-2 with C-1/ C-3/C-4/C-6; H-11 with C-6/C-7/C-8/C-13; H-13 with C-7/C-11/C-12 and H-14 with C-1/C-9/C-10. The H-1/H-6 trans-relationships was determined considering the NMR signal of H-1 (*δ*_H_ 1.53, td, *J* = 10.8, 3.1 Hz), which indicated diaxial interactions of this hydrogen with H-6 and H-2β, and an axial-equatorial coupling with H-2α. The configuration at C-10 was resolved take into consideration the resonance of the C-14 methyl at *δ*_C_ 21.0, which is typical of an axial stereochemistry, because the chemical shifts described for axial and equatorial orientation of this methyl are δ_C_ 21.6 and 28.0, respectively [38]. This stereochemistry was confirmed in the NOESY spectrum with an axial correlation of H-2β with H-14.

Cadinane diterpenes of this type had been isolated from the plant *Leucanthemopsis pulverulenta*, which also contains germacranolides of heliangolide type [39,40]. Later, it was suggested that the rearrangement of these lactones could led to the formation of the cadinane derivatives, which was confirmed making the same transformation by treatment with BF_3_Et_2_O [41]. Now, regarding the rearrangement described in this work, we must highlight the very mild conditions and the good specificity with which it has taken place.

### 2.3. Biological Activity of 3α-hydroxypelenolide and Biotransformed Products ***9*** and ***10***

The insecticidal, antifeedant and phytotoxic effects of compound **3** and compounds obtained by its biotransformation (**9** and **10**), were studied.

Compound **3** is a strong antifeedant against *Leptinotarsa decemlineata* with an EC_50_ value of 0.043 μg/cm^2^ (0.003-0.61, 95% Confidence Limits). This compound also showed moderate antifeedant effects against *Myzus persicae* (64.5 %SI, EC_50_ = 16.5 µg/cm^2^) [6]. The biotransformation products, **9** and **10**, were not antifeedant (%FI < 50). None of these compounds were phytotoxic against *Lactuca sativa* (100% germination at 48h and root length inhibitions of 11% for **3** and 0% for **9** and **10** at the end of the experiment).

When orally injected to *Spodoptera littoralis* larvae, a moderate antifeedant postingestive effect was observed for **9** that increased for compound **10**, with values of 79 and 81% larval weight gain (ΔB) and consumption (ΔI) respect to the control, respectively, without additional toxic effects (pANCOVA2 > 0.05) The cytotoxic effects of compound **3** on the insect cells Sf9 disappeared for the two compounds obtained by biotransformation (**9** and **10**) (Table 2).

Sesquiterpene lactones present a wide range of biological activities, including cytotoxic, antitumoral, antimicrobial, insecticidal and phytotoxic [42]. In our study, the insect antifeedant cytotoxic effects of **3** decreased when the double bond between the C-1 and C-10 positions disappeared through a process of oxidation that ended with the formation of an epoxide (**9**). However, analogous of **9**, ivaxillin and eriolin isolated from *Carpesium abrotanoide*, have shown antifeedant effects to lepidopteran larvae [43].

In tests carried out with germacranolides such as costunolide, parthenolide, 1, 10-epoxicostunolide among others, on *L. sativa* seeds, it was observed that introduction of an epoxide in the molecule produced a decrease of the germination activity and an increase in radicle length. Additionally, the absence of exocyclic methylene in C-13, transformed the activity on radicle length into inhibitory activity [44]. These data agree with the results obtained in our case, since the introduction of the epoxy (**9**) eliminated the low inhibitory activity on radicle of compound **3**.

Cadinane (**10**), with eudesmanolide structure, was the only one that showed postingestive effects. Compound **10** is an analog of artemisinin acid, a compound isolated from *A. annua* and used for the artemisinin semi-synthesis. Cadinane-type sesquiterpenes showed insecticidal and ixodicidal effects [45]. An analogous of **10**, cadine-4,10(15)-dien-3-one, produced toxicity in adults of *Cylas formicarus* and sterility in the tick *Boophilus microplus* [46]. According to Buchanan et al. [45], the C-3 and C-4 sterochemistry of cadine-4,10 (15)-dien-3-one derivatives, have an important effect on the insecticidal properties of these compounds, decreasing the activity when the C-4 configuration is R and when the stereochemistry of the 3α-hydroxyl is reversed. Cadinane-type sesquiterpenes also showed antigermination and phytotoxic activity against lettuce and radish seeds [45,47,48]. However, **10** was not phytotoxic in our experiments.

## 3. Materials and Methods

### 3.1. General Experimental Procedures

Melting points were determined with a Reichert Thermovar apparatus and are uncorrected (Reichert Technologies, Buffalo, NY, USA). Optical rotations were determined at room temperature on a Perkin Elmer 343 polarimeter (Perkin Elmer, Waltham, MA, USA). IR spectra were taken in a Bruker IFS 66/S spectrometer. NMR spectra were run on a Bruker AMX-500 spectrometer with pulsed field gradient using the solvent (CDCl_3_) as internal standard (Bruker Corporation, Billerica, MA, USA). EIMS and exact mass measurements were recorded on a Micromass Autospec instrument at 70 eV. Preparative and semipreparative HPLC was carried out with a Beckman Coulter 125P equipped with a diode-array detector Beckman Coulter 168 (Beckman Coulter Life Sciences, Brea, CA, USA) and preparative Interstil Prep-sil 20 mm × 250 mm, 10 µm particle size (Gasukuro Kogio, Shinjuku-ku, Tokyo, Japan) and semipreparative Ultrasphere silica 10 mm × 250 mm, 5 µm particle (Beckman Coulter Life Sciences, Brea, CA, USA) size columns. Silica gel 60 F_254_ (Merck 105715, Darmstadt, Germany) and Sephadex LH-20 (Sigma-Aldrich, St. Louis, MO, USA) were used for column chromatography. Computational analysis was carried out with the Hyperchem 7.0 program applying the Polak-Ribiere minimization algorithm.

### 3.2. Plant Material

*Artemisia absinthium* L. was cultivated in an experimental field at Ejea de los Caballeros, Zaragoza, Spain, at 345 m of altitude, with exemplars obtained from natural populations at San Blas (Teruel, Spain). A detailed description of the cultivar has been described [49]. Vegetal material was collected in flowering state, which was subjected to a drying process in the shade, with air flow, for eight days.

### 3.3. Extraction and Isolation

Dry plant (3.5 kg) was left in maceration with acetone at room temperature for four days. The cold extract was filtered and concentrated in vacuo to afford a syrup gum (246 g).

#### 3.3.1. Biodirected Chemical Fractionation

Depending on the levels of activity against different target insects, fractionation guided by bioassays was carried out, obtaining the pure compounds responsible for the activity. Of the different fractions, only those with significant biological activity were analyzed. Chromatography of the extract on a vacuum liquid column (VLC) (22 × 10 cm) eluting with a gradient of increasing polarity of Hex:EtOAc, following Coll and Bowden [50] recommendations, afforded 49 fractions being biologically active the following:

Fractions 10–11 (Hex:EtOAc 3:1): dehydroabietic acid (1.0 mg), 24-methylencycloartanol (6.7 mg), 24-methylencycloartanol acetate (1.1 mg), lagerenol (6.8 mg), lagerenol acetate (4.9 mg), cycloart-23(Z)-ene-3β,25 diol (7.1 mg), ketopelenolide A (**4**) (29.2 mg), ketopelenolide B (**5**) (5.2 mg), dihydrocostunolide (**6**) (3.6 mg), espeletone (**11**) (7.7 mg), ajenjol (**12**) (11 mg), a benzofuran derivative (**19**) (1.6 mg), β-sitosterol, campesterol and stigmasterol. Fraction 13 (Hex:EtOAc 3:1): absilactone (**1**) (19.5 mg), hansonlactone (**2**) (4.0 mg) and 3α-hydroxipelenolide (**3**) (494 mg). Fraction 14 (Hex:EtOAc 3:1): (-)-(3*S*, 5*Z*)-2,6-dimethylocta-5,7-dien-2,3-diol (25.2 mg) and artemetine. Fraction 16 (Hex:EtOAc 1:1): artemetine (214 mg) and casticine (131 mg). Additional chromatographies of these main fractions on medium pressure Si gel columns, Sephadex LH-20 and preparative HPLC were necessary to isolate these compounds.

#### 3.3.2. Absilactone (**1**)

M.p. 113–115 °C (Hex/EtOAc), [α]_D_ = −87.6 (c = 0.29, CHCl_3_); IR: ν_max_ (CCl_4_) 3403, 2931, 1773; ^1^H NMR (500 MHz): *δ* 1.25 (3H, d, *J* = 7.0 Hz, H-13), 1.65 (1H, m, H-8β), 1.95 (1H, ddd, *J* = 10.5, 3.2 Hz, H-7), 2.14 (1H, m, H-8α), 2.23 (3H, s, H-15), 2.31 (1H, dd, *J* = 12.3, 7.0 Hz, H-11), 2.36 (3H, s, H-14), 2.48 (1H, m, H-9α), 2.74 (1H, dt, *J* = 18.5, 5.6 Hz, H-9β), 4.85 (1H, br dd, *J* = 10.6, 1.6 Hz, H-6); MS *m/z* (rel. int.): 248 [M]^+^ (100), 205 (10), 177 (18), 175 (19), 161 (20), 91 (15), 86 (11), 84 (16); HRMS: [M]^+^ *m/z* at 248.1040. Calculated for C_14_H_16_O_4_, 248.1049.

#### 3.3.3. Hansonlactone (**2**)

^1^H NMR (500 MHz, CDCl_3_): *δ* 1.20 (3H, d, *J* = 7.0 Hz, H-13), 1.26 (3H, s, H-14), 1.60 (1H, m, H-8β), 1.89 (1H, t, *J* = 11.8 Hz, H-7), 1.91 (1H, m, H-9α), 2.09 (3H, d, *J* = 1.5 Hz, H-15), 2.24 (1H, m, H-11), 2.32 (1H, dd, *J* = 14.6, 8.5 Hz, H-9β), 2.59 (1H, m, H-8α), 5.00 (1H, dt, *J* = 11.8, 1.9 Hz, H-6), 5.69 (1H, dd, *J* = 7.0, 1.5 Hz, H-3), 6.37 (1H, d, *J* = 7.0 Hz, H-2); MS *m/z* (rel. int.): 262 [M]+ (12), 234 (24), 220 (13), 219 (100), 190 (13), 175 (17), 161 (9), 149 (25), 137 (23), 122 (11); HRMS: [M]^+^ at *m/z* 262.1212. Calculated for C_15_H_18_O_4_, 262.1205.

#### 3.3.4. 3α-hydroxypelenolide (**3**)

^1^H NMR (500 MHz, CDCl_3_): *δ* 0.98 (3H, d, *J* = 7.1 Hz, H-15), 1.13 (3H, d, *J* = 7.5 Hz, H-13), 1.49 (1H, dtd, *J* = 14.0, 12.6, 2.8 Hz, H-8), 1.64 (1H, dtd, *J* = 14.0, 4.1, 2.0 Hz, H-8), 1.67 (3H, s, H-14), 1.85 (3H, br s, H-4 and 2H-5), 2.06 (1H, td. *J* = 12.8, 3.2 Hz, H-9), 2.14 (1H, tt, *J* = 10.2, 2.4 Hz, H-7), 2.28 (1H, m, H-9), 2.30 (1H, m, H-2), 2.41 (1H, ddd, *J* = 13.6, 10.5, 2.7 Hz, H-2), 2.77 (1H, m, H-11), 4.02 (1H, br s, H-3), 4.08 (1H, br s, H-6), 5.51 (1H, br s, H-1); MS *m/z* (rel. int.): 252 [M]^+^ (6), 235 (45), 217 (9), 189 (19), 161 (100), 83 (48).

#### 3.3.5. Espeletone (**11**)

^1^H NMR (500 MHz, CDCl_3_): *δ* 0.96 (6H, d, *J* = 6.7 Hz, H-12 and H 13), 2.22 (1H, dt, *J* = 13.4, 6.7 Hz, H-11), 2.58 (3H, s, H-8), 2.84 (2H, d, *J* = 6.8 Hz, H-10), 3.97 (3H, s, -OMe), 7.01 (1H, d, *J* = 8.7 Hz, H-3), 8.09 (1H, dd, *J* = 8.7, 2.4 Hz, H-2), 8.21 (1H, d, *J* = 2.4 Hz, H-6); MS *m/z* (rel. int.): 234 [M]^+^ (6), 219 (6), 192 (7), 178 (11), 177 (100), 119 (6); HRMS: [M]^+^ at *m/z* 234.1261. Calculated for C_14_H_18_O_3_, 234.1256.

#### 3.3.6. Ajenjol (**12**)

M.p. 86–88 °C (Hex-EtOAc); ^1^H NMR (500 MHz, CDCl_3_): *δ* 0.95 (6H, d, *J* = 6.7 Hz, H-12 and H 13), 2.21 (1H, dt, *J* = 13.4, 6.7 Hz, H-11), 2.62 (3H, s, H-8), 2.81 (2H, d, *J* = 6.8 Hz, H-10), 3.94 (3H, s, -OMe), 6.45 (1H, s, H-3), 8.25 (1H, s, H-6), 12.92 (1H, s, -OH); MS *m/z* (rel. int.): 250 [M]^+^ (20), 235 (10), 208 (20), 194 (22), 193 (100), 175 (11); HRMS: [M]^+^ at *m/z* 250.1201. Calculated for C_14_H_18_O_4_, 250.1205.

#### 3.3.7. A Benzofuran Derivative (**19**)

^1^H NMR (500 MHz, CDCl_3_): *δ* 1.69 (6H, s, H-11 and H-12), 2.65 (3H, s, H-14), 6.66 (1H, d, *J* = 0.95 Hz, H-3), 7.49 (1H, d, *J* = 8.6 Hz, H-7), 7.93 (1H, dd, *J* = 8.6, 1.8 Hz, H-6), 8.18 (1H, d, *J* = 1.8 Hz, H-4); MS *m/z* (rel. int.): 218 [M]^+^ (27), 203 (100), 200 (10), 185 (19), 175 (3), 160 (6), 157 (6), 128 (4); HRMS: [M]^+^ at *m/z* 218.0492. Calculated for C_13_H_14_O_3_, 218.0493.

### 3.4. Biotransformation and Rearrangement

#### 3.4.1. Microorganism

The fungal strain, *Mucor plumbeus* CMI 116688, was a gift from Prof. J.R. Hanson, Department of Chemistry, University of Sussex, UK.

#### 3.4.2. Incubation of 3α-hydroxypelenolide (**3**)

*M. plumbeus* was grown in shake culture, at 25 °C, under light and stirring at 120 rpm, for two days in 18 conical flasks (500 mL), each containing sterile medium (100 mL) comprising (per dm^3^) glucose (80 g), NH_4_NO_3_ (0.48 g), KH_2_PO_4_ (5 g), MgSO_4_·7H_2_O (1 g) and trace elements solution (2 mL). The trace elements solution contained (per 100 mL) Co (NO_3_)_2_·6H_2_O (0.01 g), CuSO_4_·5H_2_O (0.015 g), ZnSO_4_·7H_2_O (0.16 g), MnSO_4_·4H_2_O (0.01 g), FeSO_4_·7H_2_O (0.1 g) and (NH_4_)_6_Mo_7_O_24_·4H_2_O (0.01 g). The substrate 3 (315 mg) in EtOH (3.0 mL) and Tween 80 (0.2 mL) was distributed equally between the flasks and the incubation allowed to continue for a further six days. The mycelium was filtered, and the culture filtrate was extracted with EtOAc in a soxhlet. The solvent was evaporated to afford a residue, which was chromatographed on a silica gel column using a Hex-EtOAc and EtOAc-MeOH gradients to give substrate **3** (145 mg, Hex-EtOAc 20%), epoxide **9** (46 mg, EtOAc) and the rearranged product **10** (94 mg). EtOAc-MeOH, 50%).

#### 3.4.3. 1β,10α-Epoxy-3α-hydroxypelenolide (**9**)

M.p. 164-166 °C (Hex-EtOAc); [α]_D_ = - 49 (c = 0.21, CHCl_3_); ^1^H NMR (500 MHz, CDCl_3_, 233 ºK): *δ* 1.06 (3H, d, *J* = 7.2 Hz, H-15), 1.10 (1H, m, H-9α), 1.15 (3H, d, *J* = 7.0 Hz, H-13), 1.31 (1H, s, H-14), 1.37 (1H, m, H-8β)), 1.42 (1H, m, H-5β), 1.66 (1H, dd, *J* = 17.1, 2.0 Hz, H-8α), 1.76 (2H, m, H-2 and H-4), 2.15 (1H, br t, *J* = 11.2 Hz, H-7), 2.25 (2H, m, H-2 and H-5 α), 2.38 (1H, dd, *J* = 13.2, 4.0 Hz, H- 9β), 2.84 (1H, m, H-11), 3.28 (1H, br d, *J* = 8.5 Hz, H-1), 4.01 (1H, br d, *J* = 5.5 Hz, H-3), 4.26 (1H, br d, *J* = 11.4 Hz, H-6); MS *m/z* (rel. int.) 268 [M]^+^ (1), 250 (2), 240 (4), 223 (3), 194 (28), 182 (13), 179 (15), 167 (7), 151 (34), 141 (12), 125 (37), 55 (100).

#### 3.4.4. 1(R), 4(S), 6(S), 7(R), 11(R)-3α, 10α-Dihydroxy-cadinan-12-oic acid (**10**)

Rearrangement of the substrate **3** in the culture medium produced compound **10**, which was confirmed in an experiment carried out with the incubation of **3** (35 mg) in the culture medium (200 mL) in the same conditions but without fungus; ^1^H NMR (500 MHz, CD_3_OD): *δ* 0.94 (3H, d, *J* = 6.9 Hz, H-15), 1.00 (1H, br t, *J* = 12.6 Hz, H-5α), 1.04 (3H, s, H-14), 1.11 (3H, d, *J* = 7.1 Hz, H-13), 1.22 (1H, td, *J* = 13.0, 2.5 Hz, H-2β), 1.28 (1H, td, *J* = 11.1, 3.3 Hz, H-6), 1.35 (2H, m, H-7 and H-8), 1.46 (2H, m, H-9 and H-4), 1.51 (1H, td, *J* = 10.8, 3.1 Hz, H-1), 1.74 (3H, m, H-5β. H-8 and H-9), 2.09 (1H, dt, *J* = 13.5, 2.4 Hz, H-2α), 2.77 (1H, ddd, *J* = 14.2, 7.1, 2.0 Hz, H-11), 3.81 (1H, dd, *J* = 5.5, 2.6 Hz, H-3); MS *m/z* (rel. int.) 255 (M-15)^+^ (18), 252 (6), 237 (50), 234 (14), 219 (8), 197 (30), 179 (60), 178 (68), 161 (100), 145 (47), 137 (32), 121 (69); HRMS *m/z* 255.1588. Calculated for C_14_H_23_O_4_, 255.1596.

### 3.5. Insect Bioassays

*Spodoptera littoralis* were reared on artificial diet while *Leptinotarsa decemlineata* and *Myzus persicae* colonies were reared on their host plants (*Solanum tuberosum* and *Capsicum annuum*), and maintained at 22 ± 1 °C, >70% relative humidity with a photoperiod of 16:8 h (L:D) in a growth chamber.

#### 3.5.1. Choice Feeding Assays

These were conducted with newly emerged *S. littoralis* sixth-instar larvae, *L. decemlineata* and apterous adult aphids as described [51]. The upper surface of *C. annuum* and *S. tuberosum* leaf disks or fragments (1.0 cm^2^) were treated with 10 μL of the test substance at a concentration of 50 μg/cm^2^ Six Petri dishes or twenty boxes (2 × 2 cm) with three (*S. littoralis/L. decemlineata*) or ten (aphids) insects each were allowed to feed in a growth chamber (environmental conditions as described above). Each experiment was repeated three times and terminated after the consumption of between 50–75% of the control disks (*S. littoralis/L. decemlineata*) or after 24 h (aphids).

Feeding inhibition or aphid settling was calculated by measuring the disk surface consumption (digitalized with https://imagej.nih.gov/ij//, accessed on 20 April 2021) [52] or by counting the number of aphids on each leaf fragment. Feeding/settling inhibition (%FI or %SI) was calculated as % FI/SI = [1 − (T/C) × 100], where T and C represent feeding/settling on treated and control leaf disks, respectively. The antifeedant effects (% FI/SI) were analyzed for significance by the nonparametric Wilcoxon paired signed-rank test comparing the consumption/settling between the treatment and control leaf disks. Extracts and compounds with an SI > 60% were further tested in a dose-response experiment (1:2 serial dilutions to cover a range of activities between 100 and <50% feeding inhibition with a minimum of 3 doses) to calculate their relative potency (EC_50_, the effective dose to give a 50% settling reduction) from linear regression analysis (% FI/SI on Log-dose, STATGRAPHICS Centurion XVI, version 16.1.02).

#### 3.5.2. Oral Cannulation

It was performed with pre-weighed newly emerged *S. littoralis* L6-larvae. Each experiment consisted of twenty larvae orally dosed with 40 µg of the test compound in 4 µL of dimethyl sulfoxide (DMSO). Negative controls received 5 µL of DMSO. At the end of the experiment (72 h), larval consumption and growth were calculated on a dry-weight basis. An analysis of covariance (STATGRAPHICS Centurion XVI, version 16.1.02) (ANCOVA1) on biomass gains with initial biomass as covariate (covariate *p* > 0.05) was performed to test for significant effects of the test compounds. A second analysis (ANCOVA2) was performed on biomass gains with food consumption as covariate to test for postingestive effects [51].

### 3.6. Cytotoxicity

Sf9 cells derived from *Spodoptera frugiperda* pupal ovarian tissue (European Collection of Cell Cultures, ECCC) were maintained in TC-100 insect cell medium supplemented with 10% fetal bovine serum, 1% L-glutamine and 1% penicillin/streptomycin at 26 °C. Cells seeded in 96-well flat-bottom microplates with 100 μL medium per well, were exposed for 48 h to serial dilutions of the test compounds in DMSO (<1% final concentration). Cell viability was analyzed by the MTT (3-(4,5-dimethylthiazol-2-yl) -2,5-diphenyltetrazolium bromide) colorimetric assay method and the purple-colored formazan precipitate was dissolved with 100 μL of DMSO as described by González-Coloma et al. [53]. The active compounds were tested in a dose-response experiment to calculate their relative potency (IC50) values, the effective dose to give 50% cell viability, which was determined from linear regression analysis (% cell viability on log dose).

### 3.7. Phytotoxicity

These experiments were conducted with *Lactuca sativa* var. Carrascoy seeds as described by [54]. The germination was monitored daily for 6 days and the radicle length measured at the end of the experiment (20 roots randomly selected for each experiment digitalized with https://imagej.nih.gov/ij//, accessed on 20 April 2021) [52]. A non-parametric analysis of variance (ANOVA) was performed on germination and radicle length data (STATGRAPHICS Centurion XVI, version 16.1.02).

## 4. Conclusions

Three new compounds, the sesquiterpenes absilactone and hansonlactone and the acetophenone derivative ajenjol, have been isolated from a cultivated variety of *Artemisia absinthium*, along with several known ones ((-)-(3S,5Z)-2,6-dimethylocta- -5,7-dien-2,3-diol, the sesquiterpene lactones 3α-hydroxypelenolide, ketopelenolide A, ketopelenolide B and dihydrocostunolide, the diterpene dihydroabietic acid, the triterpenes 24-methylenecycloartanol and its acetate, lagerenol and its acetate, and cycloart-23(Z)-en-3β,25-diol, the acetophenone espeletone, a benzofuran derivative, and the flavones artemetin and casticine).

The major compound **3**, 3α-hydroxypelenolide, was a strong antifeedant against *Leptinotarsa decemlineata* and moderate antifeedant against *Myzus persicae*. Its biotransformation products, **9** and **10**, were not antifeedant. None of these compounds were phytotoxic against *Lactuca sativa*. When orally injected to *Spodoptera littoralis* larvae, a moderate antifeedant postingestive effect was observed for **9** that increased for compound **10**. The cytotoxic effects of compound **3** on the insect cells Sf9 disappeared for the two compounds obtained by biotransformation (**9** and **10**).

## Figures and Tables

**Table 1:** ^13^C NMR data of compounds **1**–**3**, **9**–**12**, **14** and **19**.

Carbon	1	2	3	9	10 ^a^	11	12	14	19
1	121.2	168.8	123.3	59.0	45.8	128.9	113.8	128.5	-
2	166.8	138.8	34.4	34.1	34.4	133.2	167.5	136.1	164.8
3	-	117.3	73.6	71.3	71.2	111.5	99.7	118.7	100.9
4	149.0	127.9	40.2	38.0	37.5	161.6	164.8	166.3	122.3
5	111.6	129.6	38.6	36.0	33.9	130.2	120.9	119.2	132.8
6	78.3	80.1	86.1	85.3	42.1	131.1	135.1	131.2	124.9
7	49.0	45.3	47.3	47.0	47.9	196.4	203.4	195.7	111.2
8	26.0	23.2	27.3	24.0	26.2	26.4	26.2	26.3	157.4
9	37.6	32.0	40.4	39.3	43.4	202.0	199.4	206.8	128.5
10	154.4	48.0	135.8	60.5	72.8	52.6	52.6	47.0	69.4
11	41.5	42.5	37.0	37.0	40.6	24.9	24.9	25.3	28.8
12	177.6	178.4	179.4	180.2	179.5	22.7	22.7	22.7	28.8
13	12.4	12.6	10.7	10.4	15.2	22.7	22.7	22.7	197.6
14	22.6	19.8	16.3	17.0	21.0	55.9	55.9		26.7
15	13.5	21.3	19.3	22.1	19.2		-		-

^a ^Solvent: CD_3_OD.

**Table 2:** Biomass gain (ΔB) and consumption (ΔI) (expressed as percentage of the control, %C) of orally injected *Spodoptera littoralis* larvae (20 μg/insect, 20 larvae) and cytotoxic effects on insect line cells Sf9.

Compound	S. littoralis		pANCOVA2 (ΔI covariate)	Sf9 EC_50_ [μg/mL]
	ΔB ^a^	ΔI ^b^		
**3**	97	94		29.5 (19.2, 45.5)
**9**	87 *	92		>100
**10**	79 *	81 *	*p* = 0.51	>100

^a^ △B = change in insect body weight (dry weight, mg). ^b^ △I = mg food consumed (dry weight, mg). * Treatment *p* level <0.05, ANCOVA1 (initial larval weight as covariate). EC_50_ = concentration needed to produce 50% cell viability).

## Data Availability

The data presented in this study are available in the Research Group databases, within the article and its supplementary material.

## Supplementary Materials

The following are available online at https://www.mdpi.com/article/10.3390/plants10050891/s1, Figure S1: ^1^H-NMR of compound **1**; Figure S2. ^13^C-NMR of compound **1**; Figure S3. ^1^H-NMR of compound **2**; Figure S4. ^13^C-NMR of compound **2**; Figure S5. ^1^H-NMR of compound **3**; Figure S6. ^13^C-NMR of compound **3**; Figure S7. ^1^H-NMR of compound **9**; Figure S8. ^13^C-NMR of compound **9**; Figure S9. ^1^H-NMR of compound **10**; Figure S10. ^13^C-NMR of compound **10**; Figure S11. ^1^H-NMR of compound **11**; Figure S12. ^13^C-NMR of compound **11**; Figure S13. ^1^H-NMR of compound **12**; Figure S14. ^13^C-NMR of compound **12**; Figure S15. ^1^H-NMR of compound **19**; Figure S16. ^13^C-NMR of compound **19**.
