# Supplementary material for: Sesquiterpene Lactones from Artemisia absinthium. Biotransformation and Rearrangement of the Insect Antifeedant 3α-hydroxypelenolide
Source: Plants (Basel). 2021 Apr 28;10(5):891. doi: 10.3390/plants10050891 (PMC8146418; doi:10.3390/plants10050891)
Supplement: Supplementary file 1 [file plants-10-00891-s001.zip › plants-1196488-supplementary.pdf]

Sesquiterpene lactones from *Artemisia absinthium*. Biotransformation and rearrangement of 3α-hydroxypelenolide

Braulio M. Fraga, [1] Carmen E. Díaz,*[1] María Bailén,[2] Azucena González-Coloma*[3]

[1] *Instituto de Productos Naturales y Agrobiología, C.S.I.C.; Avda. Astrofísico F. Sánchez 3, 38206-La Laguna, Tenerife, Canary Islands, Spain*

[2] *Departamento de Medicina Preventiva y Salud Pública y Microbiología. Facultad de Medicina. Universidad Autónoma de Madrid. c/ Arzobispo Morcillo, s/n °- 28049, Madrid;*

[3] *Instituto de Ciencias Agrarias, CSIC, Serrano 115-dpdo, 28006 Madrid, Spain*

*Corresponding authors:

E-mail address: celisa@ipna.csic.es (C.E. Díaz)

E-mail address: azu@ica.csic.es (A. González-Coloma)

List of contents:

Figure S1. ^{1}H-NMR spectrum of compound **1** ($CDCl_3$, 500 MHz)

Figure S2. ^{13}C-NMR spectrum of compound **1** ($CDCl_3$, 125 MHz)

Figure S3. ^{1}H-NMR spectrum of compound **2** ($CDCl_3$, 500 MHz)

Figure S4. ^{13}C-NMR spectrum of compound **2** ($CDCl_3$, 125 MHz)

Figure S5. ^{1}H-NMR spectrum of compound **3** ($CDCl_3$, 500 MHz)

Figure S6. ^{13}C-NMR spectrum of compound **3** ($CDCl_3$, 125 MHz)

Figure S7. ^{1}H-NMR spectrum of compound **9** ($CDCl_3$, 500 MHz)

Figure S8. ^{13}C-NMR spectrum of compound **9** ($CDCl_3$, 125 MHz)

Figure S9. ^{1}H-NMR spectrum of compound **10** ($CDCl_3$, 500 MHz)

Figure S10. ^{13}C-NMR spectrum of compound **10** (CD_3OD, 125 MHz)

Figure S11. ^{1}H-NMR spectrum of compound **11** (CD_3OD, 500 MHz)

Figure S12. ^{13}C-NMR spectrum of compound **11** ($CDCl_3$, 125 MHz)

Figure S13. ^{1}H-NMR spectrum of compound **12** ($CDCl_3$, 500 MHz)

Figure S14. ^{13}C-NMR spectrum of compound **12** ($CDCl_3$, 125 MHz)

Figure S15. ^{1}H-NMR spectrum of compound **19** ($CDCl_3$, 500 MHz)

Figure S16. ^{13}C-NMR spectrum of compound **19** ($CDCl_3$, 125 MHz)

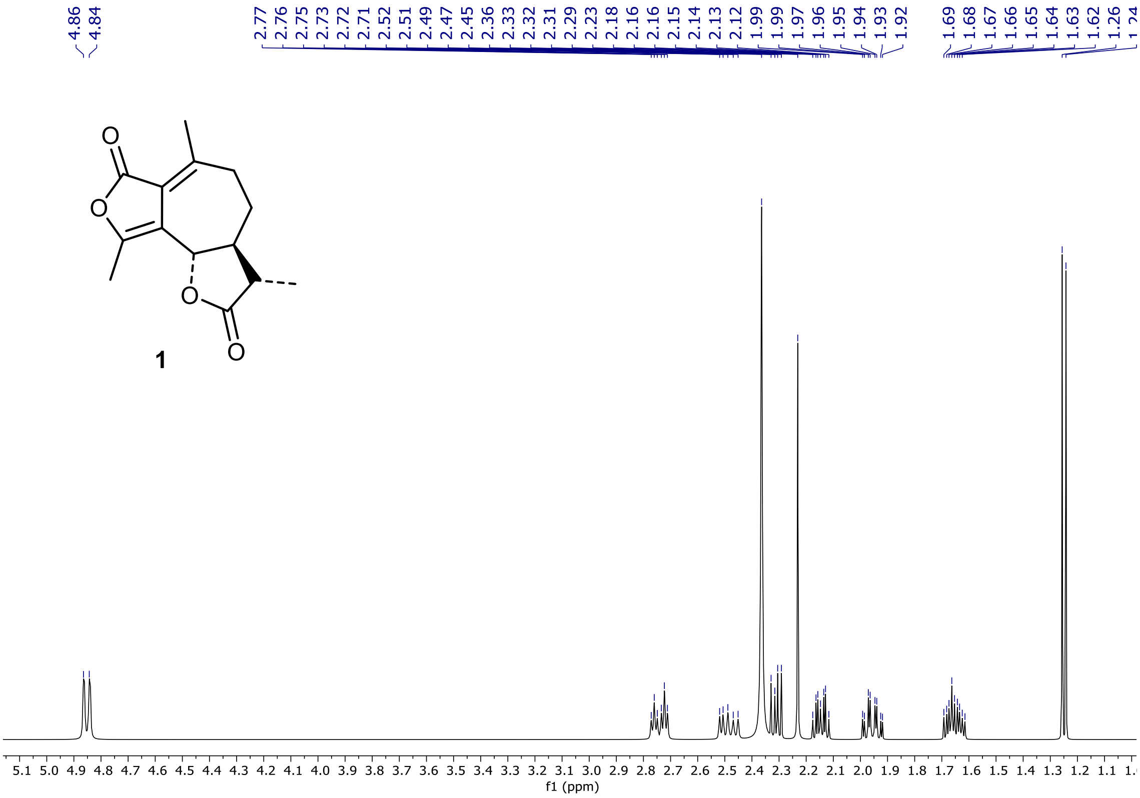

Figure S1. ^{1}H-NMR spectrum of compound **1** ($CDCl_3$, 500 MHz)

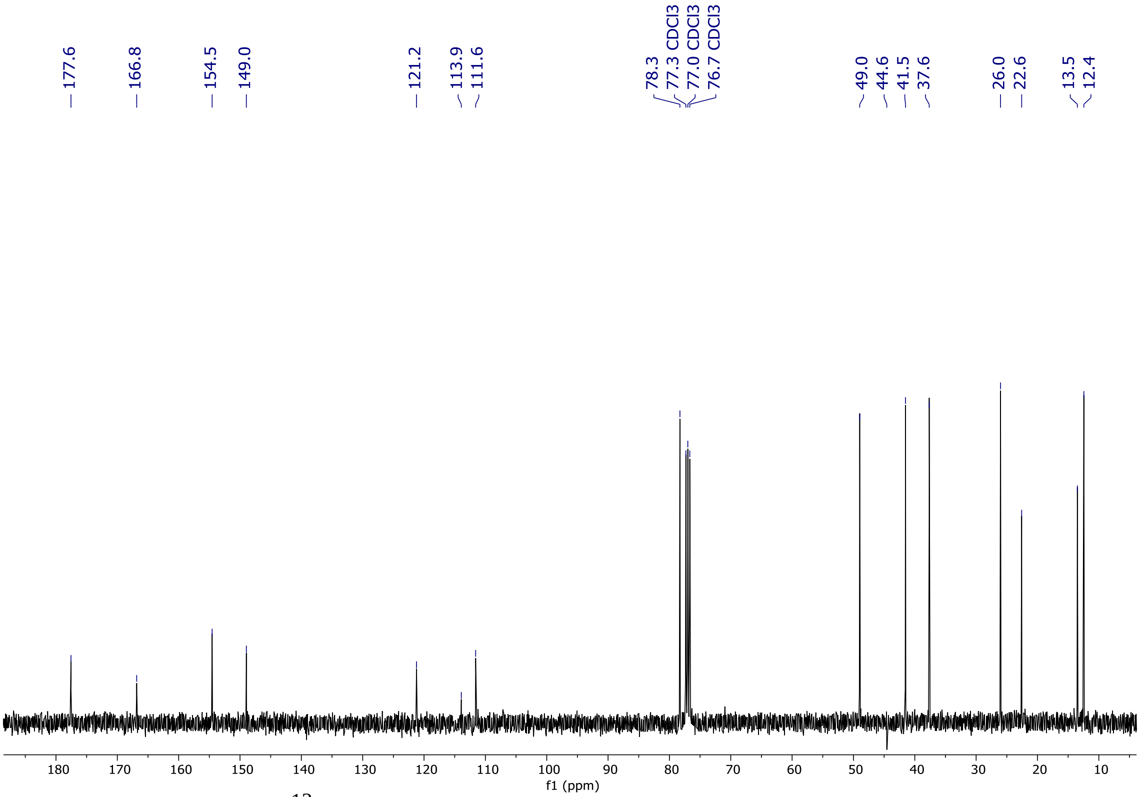

Figure S2. ^{13}C-NMR spectrum of compound **1** ($CDCl_3$, 125 MHz)

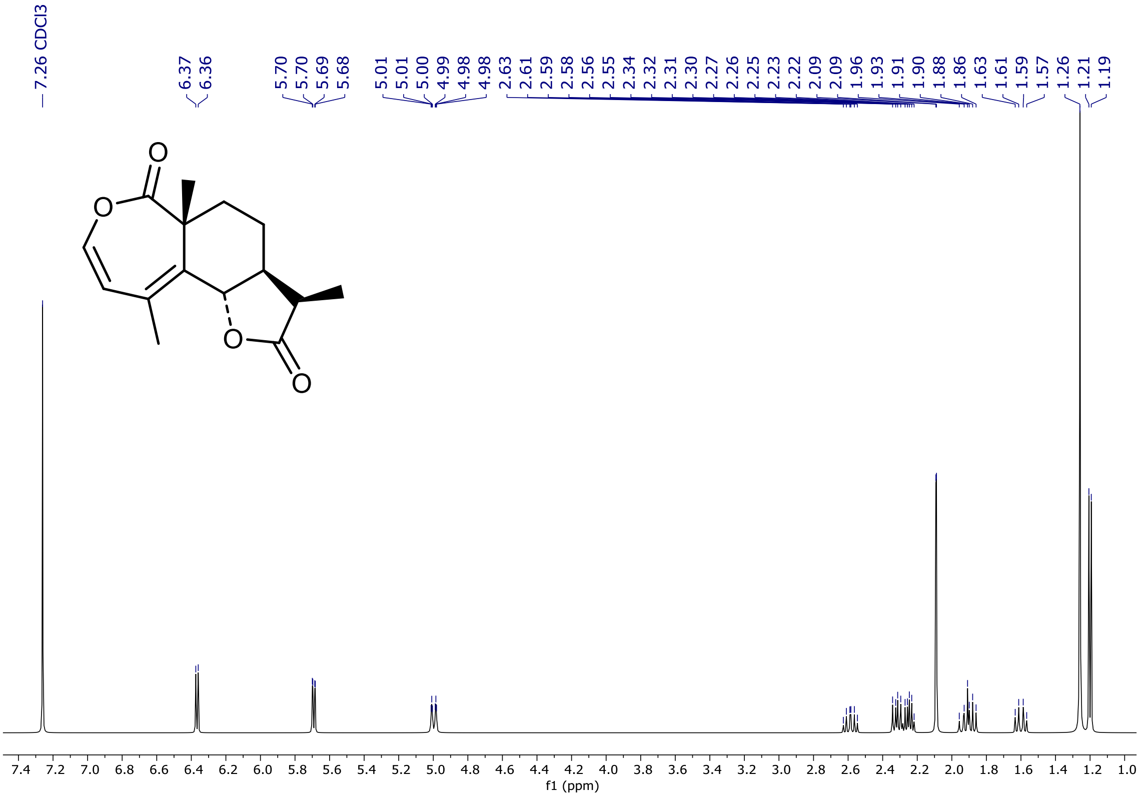

Figure S3. ^{1}H-NMR spectrum of compound **2** ($CDCl_3$, 500 MHz)

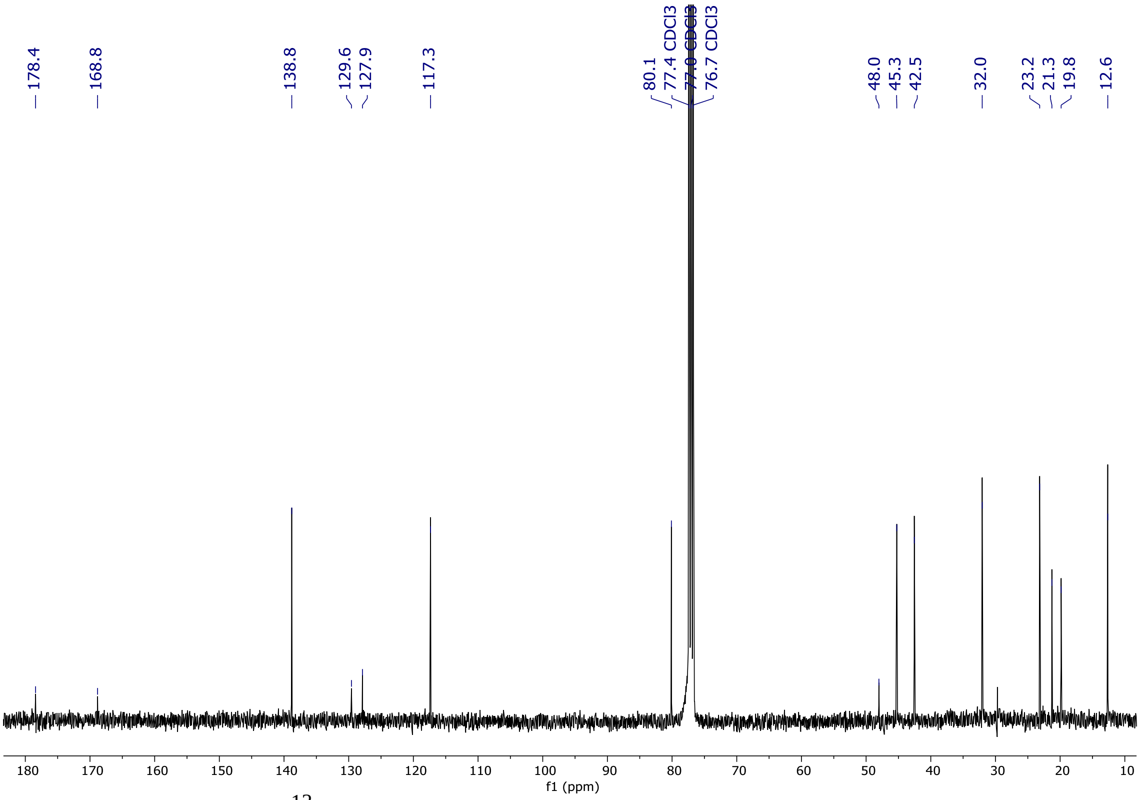

Figure S4. ^{13}C-NMR spectrum of compound **2** ($CDCl_3$, 125 MHz)

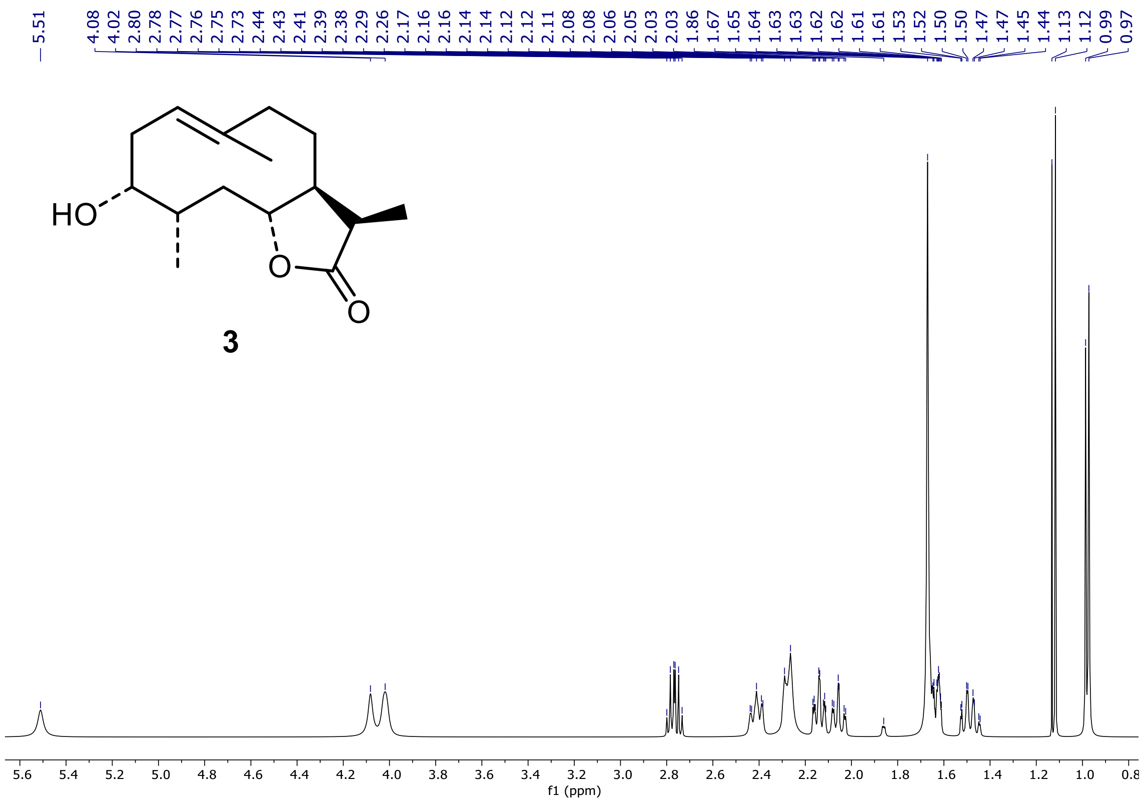

Figure S5. ^{1}H-NMR spectrum of compound **3** ($CDCl_3$, 500 MHz)

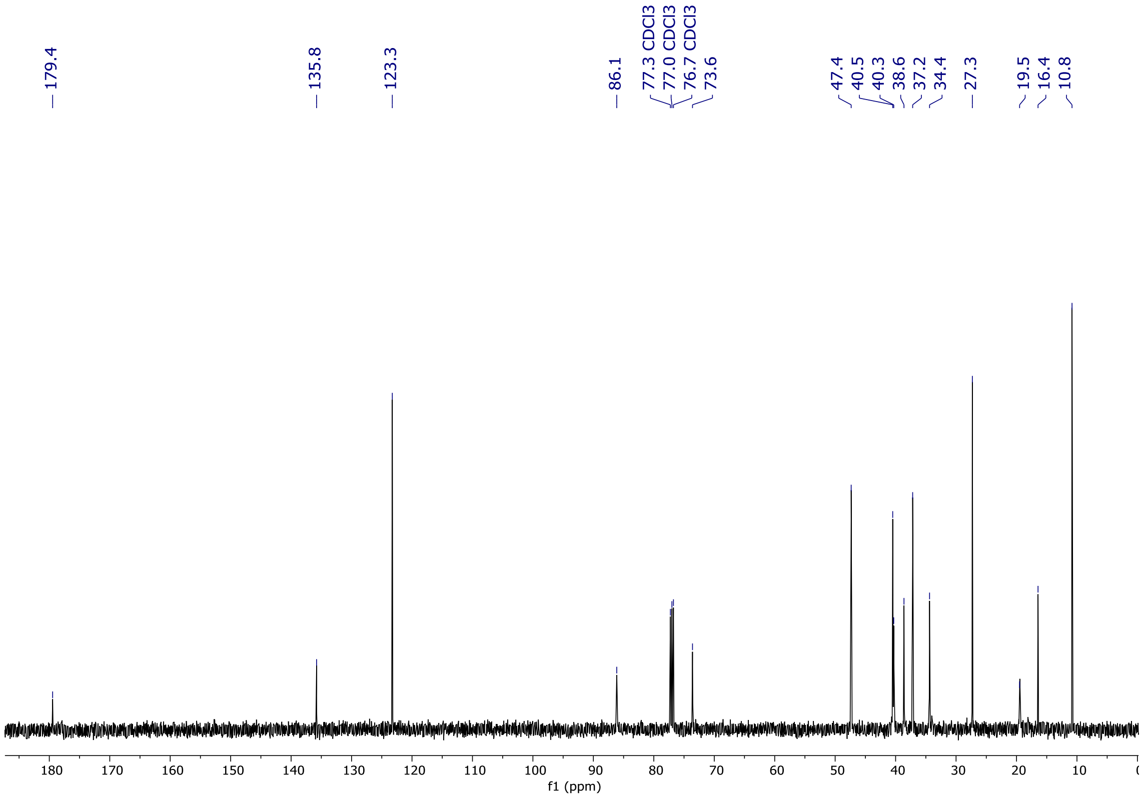

Figure S6. ^{13}C-NMR spectrum of compound **3** ($CDCl_3$, 125 MHz)

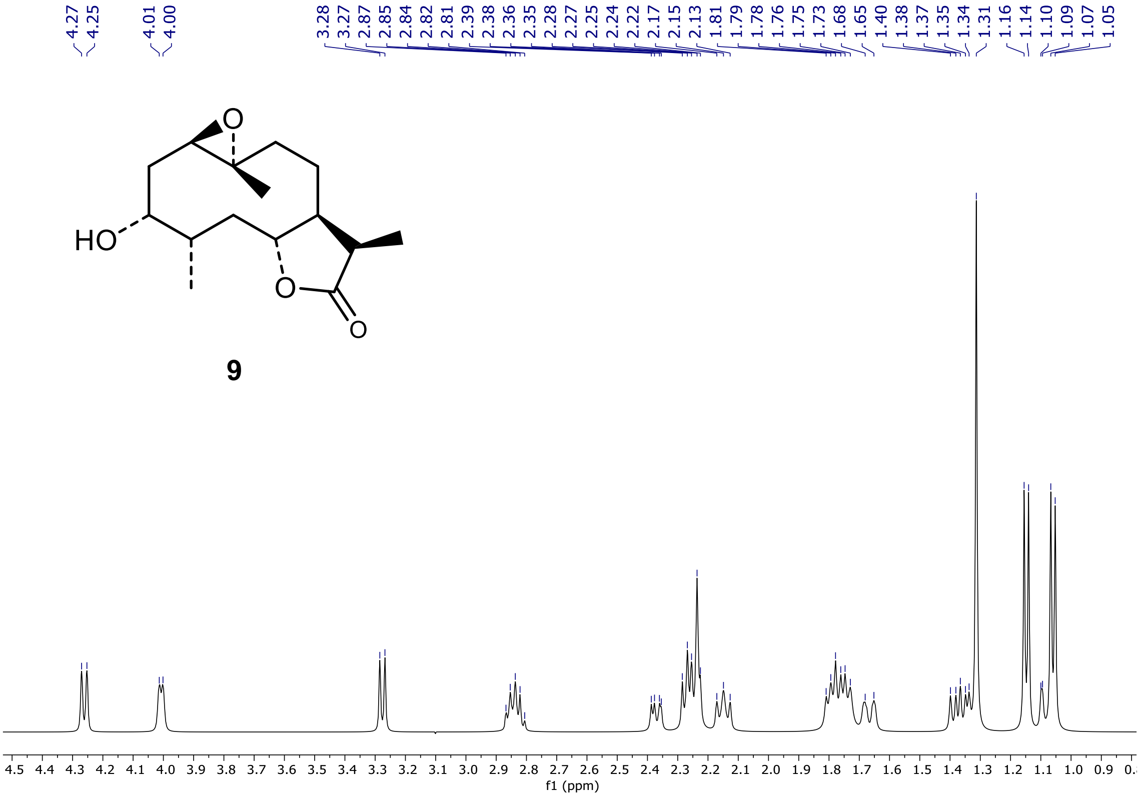

Figure S7. ^{1}H-NMR spectrum of compound **9** ($CDCl_3$, 500 MHz)

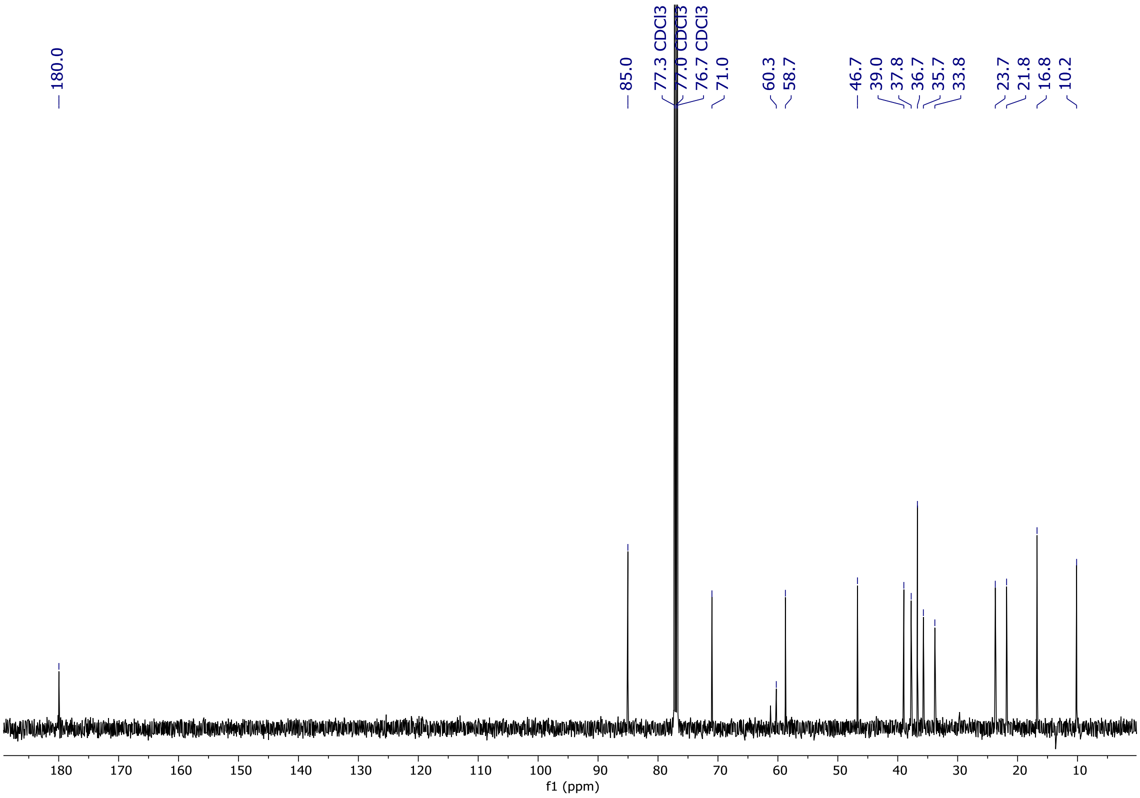

Figure S8. ^{13}C-NMR spectrum of compound **9** ($CDCl_3$, 125 MHz)

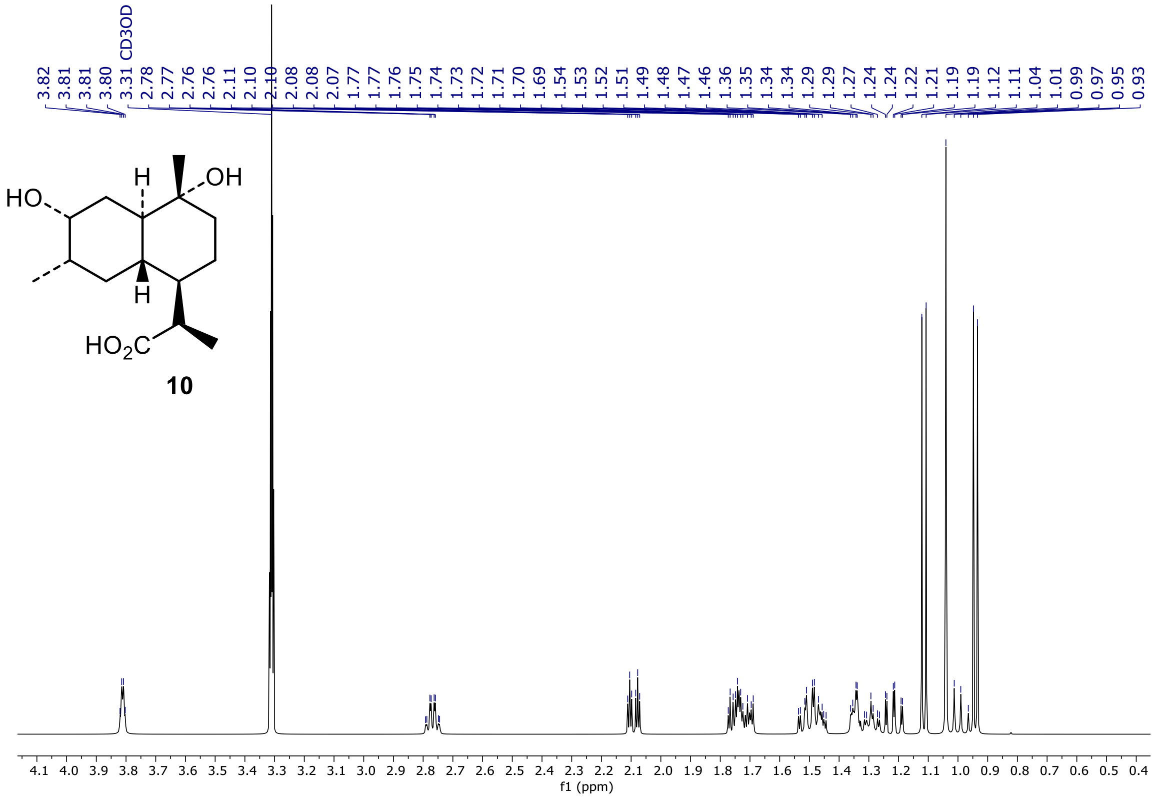

Figure S9. ^{1}H-NMR spectrum of compound **10** (CD_3OD, 500 MHz)

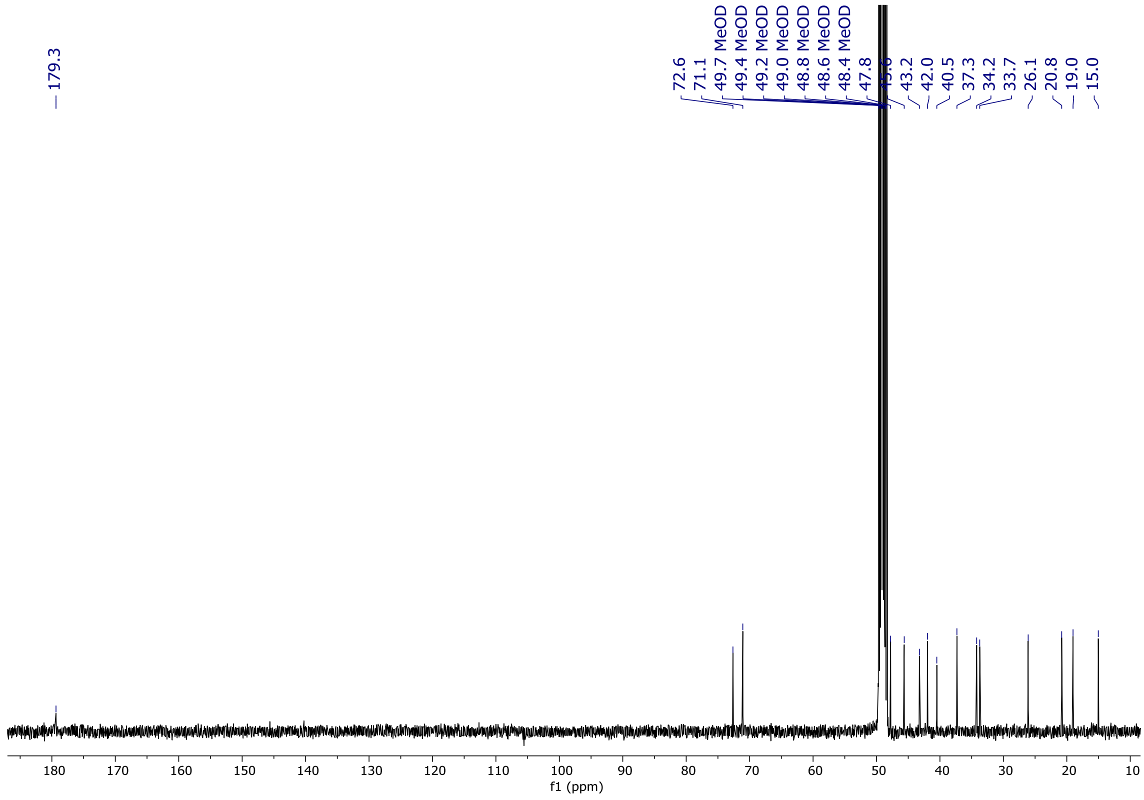

Figure S10. ^{13}C-NMR spectrum of compound **10** (CD_3OD, 125 MHz)

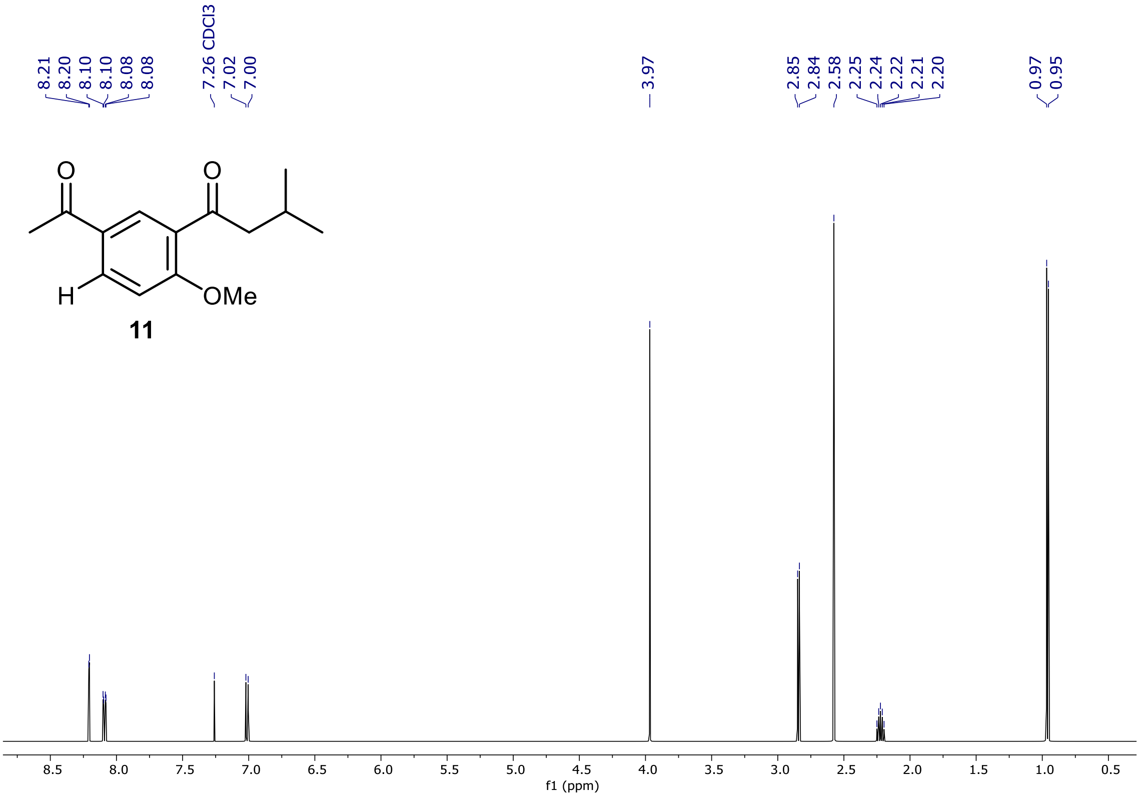

Figure S11. ^{1}H-NMR spectrum of compound **11** ($CDCl_3$, 500 MHz)

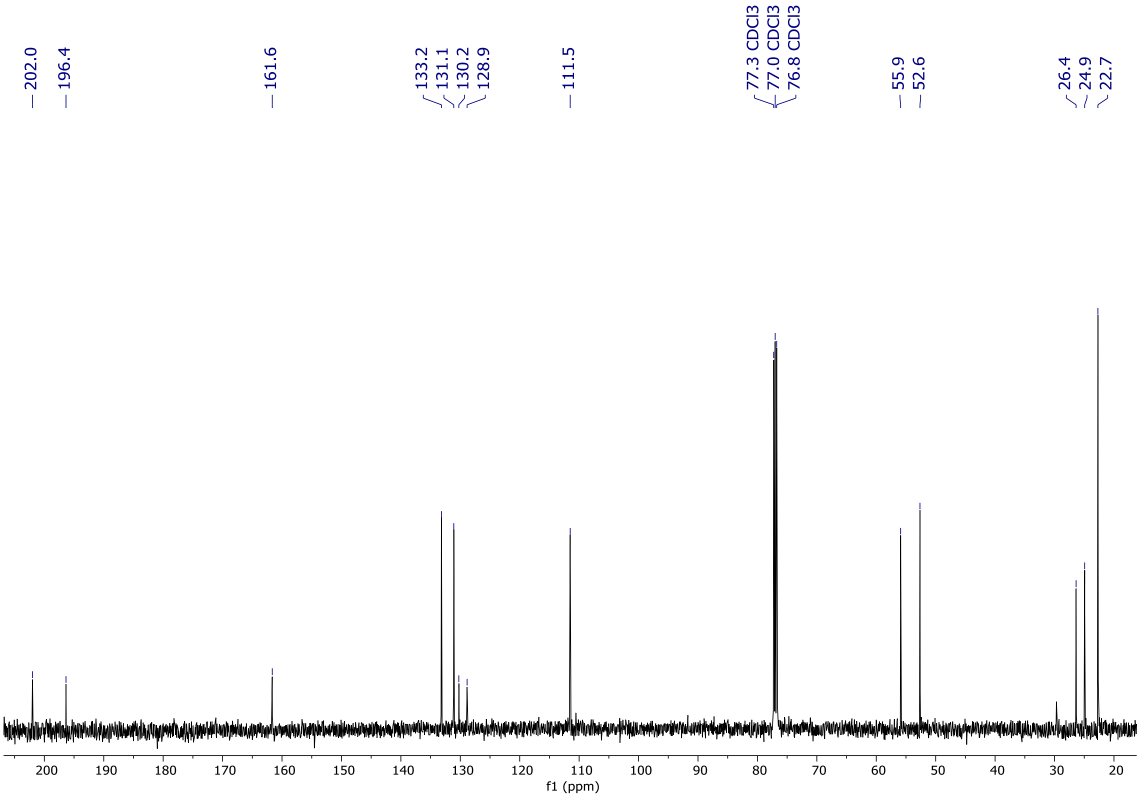

Figure S12. ^{13}C-NMR spectrum of compound **11** ($CDCl_3$, 125 MHz)

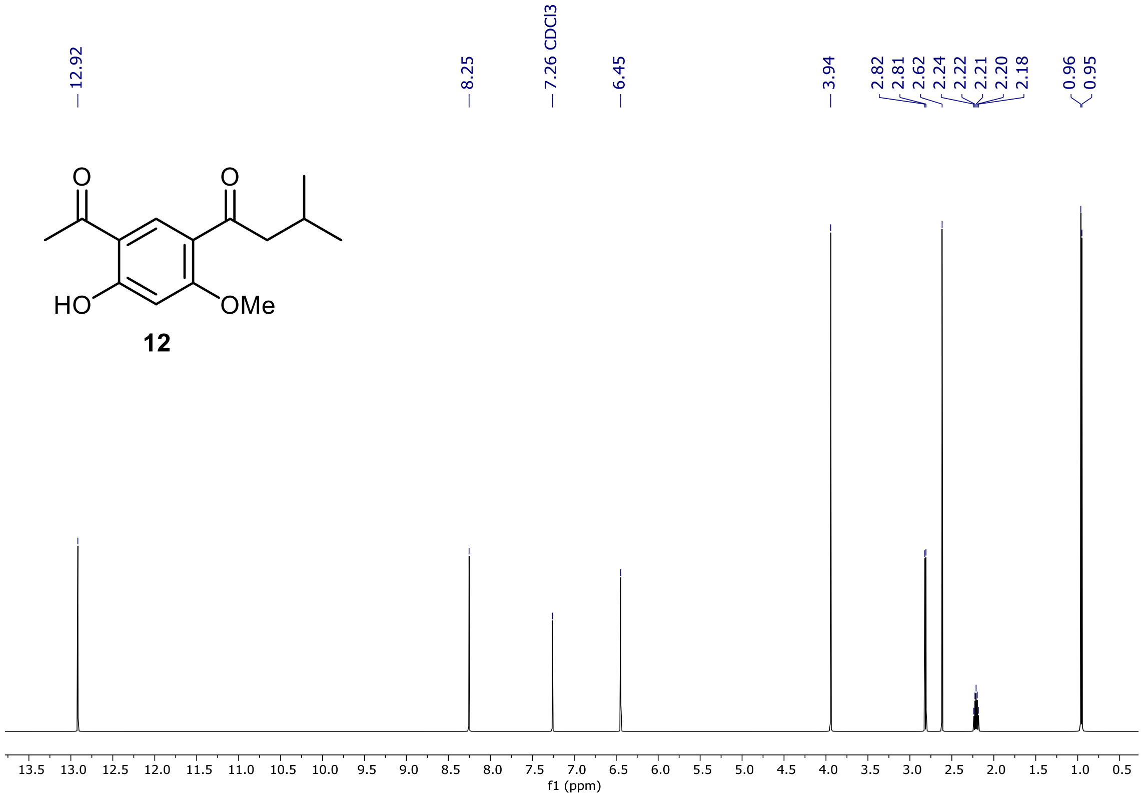

Figure S13. ^{1}H-NMR spectrum of compound **12** ($CDCl_3$, 500 MHz)

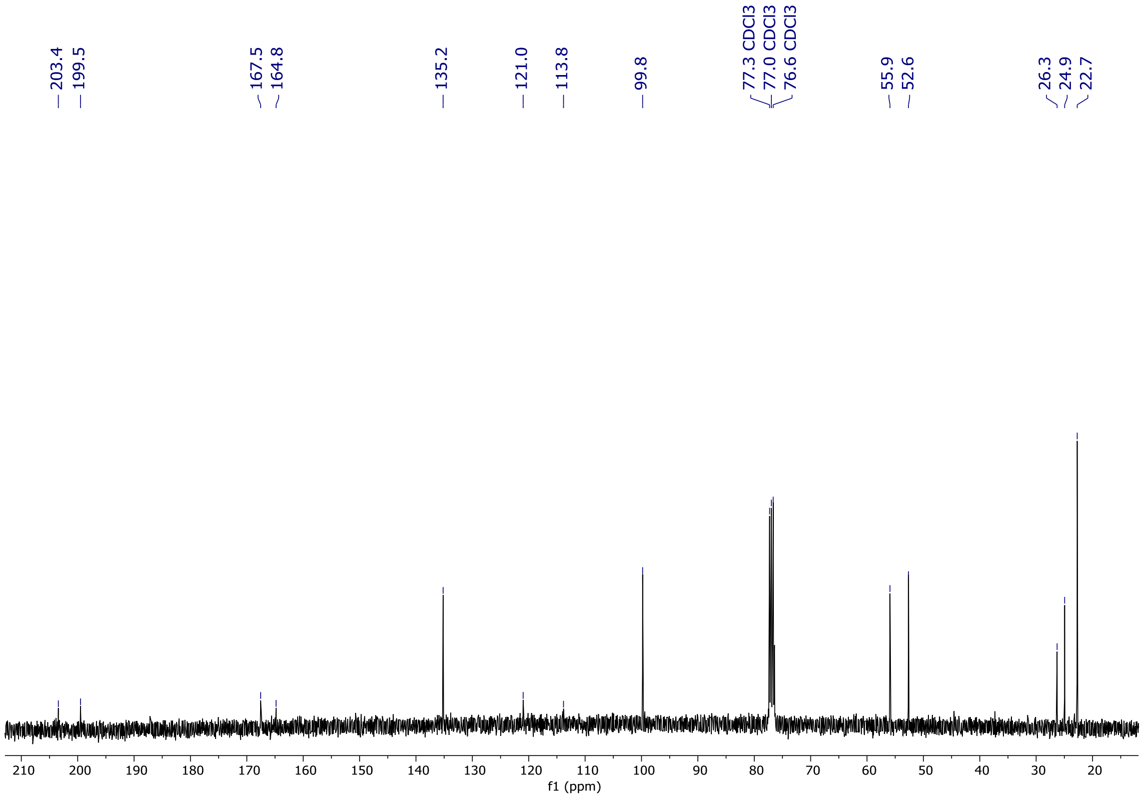

Figure S14. ^{13}C-NMR spectrum of compound **12** ($CDCl_3$, 125 MHz)

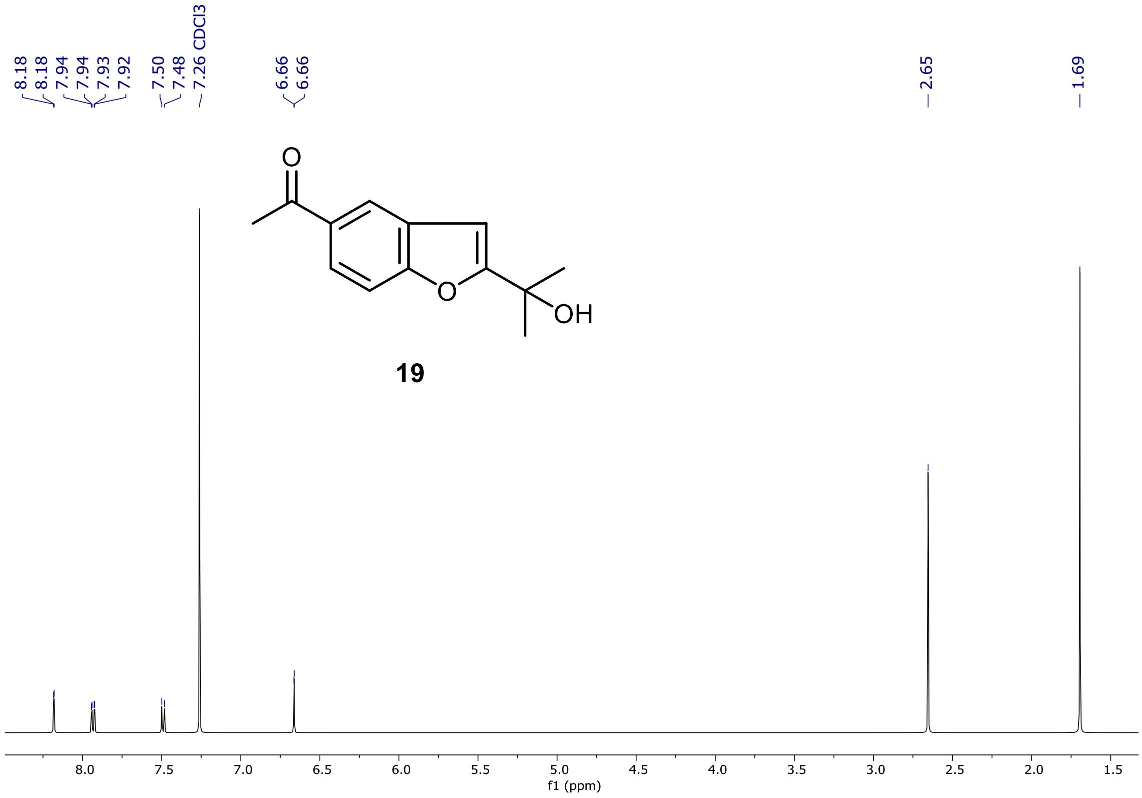

Figure S15. ^{1}H-NMR spectrum of compound **19** ($CDCl_3$, 500 MHz)

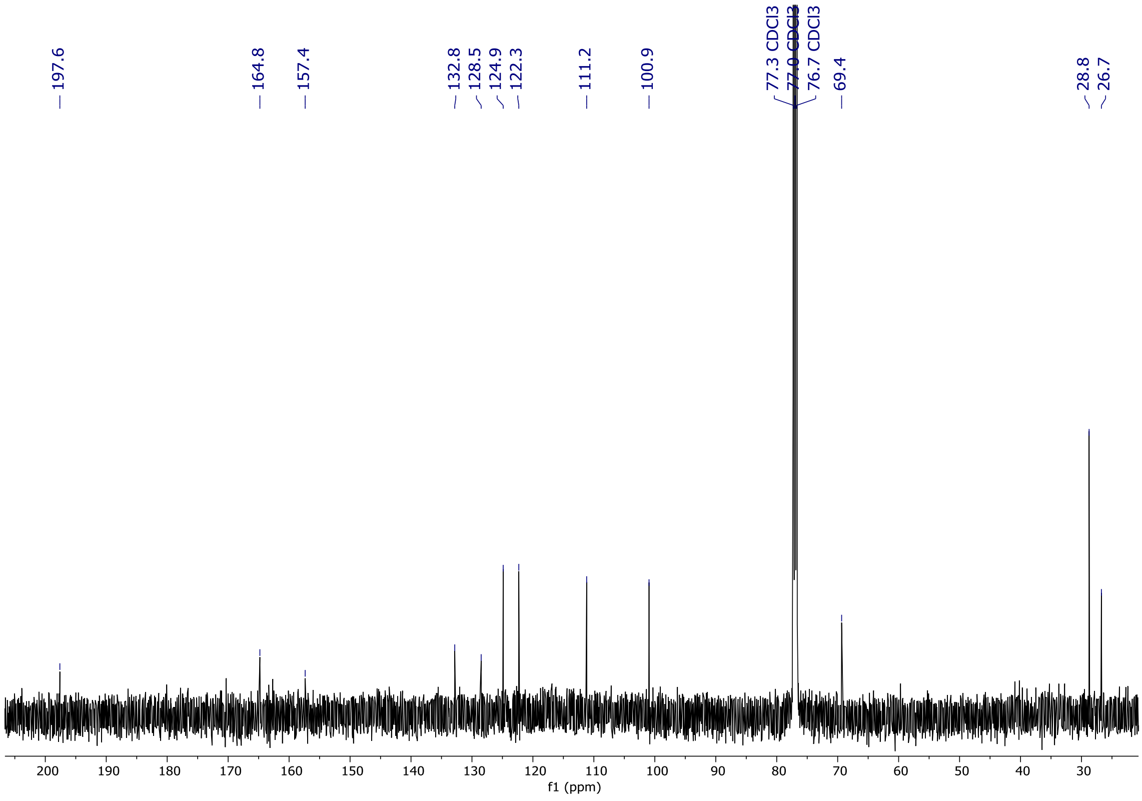

Figure S16. ^{13}C-NMR spectrum of compound **19** ($CDCl_3$, 125 MHz)
